# Precision Psychiatry Applications with Pharmacogenomics: Artificial Intelligence and Machine Learning Approaches

**DOI:** 10.3390/ijms21030969

**Published:** 2020-02-01

**Authors:** Eugene Lin, Chieh-Hsin Lin, Hsien-Yuan Lane

**Affiliations:** 1 Department of Biostatistics, University of Washington, Seattle, WA 98195, USA; lines@uw.edu; 2 Department of Electrical & Computer Engineering, University of Washington, Seattle, WA 98195, USA; 3 Graduate Institute of Biomedical Sciences, China Medical University, Taichung 40402, Taiwan; 4 Department of Psychiatry, Kaohsiung Chang Gung Memorial Hospital, Chang Gung University College of Medicine, Kaohsiung 83301, Taiwan; 5 School of Medicine, Chang Gung University, Taoyuan 33302, Taiwan; 6 Department of Psychiatry, China Medical University Hospital, Taichung 40402, Taiwan; 7 Brain Disease Research Center, China Medical University Hospital, Taichung 40402, Taiwan; 8 Department of Psychology, College of Medical and Health Sciences, Asia University, Taichung 41354, Taiwan

**Keywords:** artificial intelligence, biomarker, deep learning, machine learning, multi-omics, neural networks, neuroimaging, pharmacogenomics, precision medicine, precision psychiatry

## Abstract

A growing body of evidence now suggests that precision psychiatry, an interdisciplinary field of psychiatry, precision medicine, and pharmacogenomics, serves as an indispensable foundation of medical practices by offering the accurate medication with the accurate dose at the accurate time to patients with psychiatric disorders. In light of the latest advancements in artificial intelligence and machine learning techniques, numerous biomarkers and genetic loci associated with psychiatric diseases and relevant treatments are being discovered in precision psychiatry research by employing neuroimaging and multi-omics. In this review, we focus on the latest developments for precision psychiatry research using artificial intelligence and machine learning approaches, such as deep learning and neural network algorithms, together with multi-omics and neuroimaging data. Firstly, we review precision psychiatry and pharmacogenomics studies that leverage various artificial intelligence and machine learning techniques to assess treatment prediction, prognosis prediction, diagnosis prediction, and the detection of potential biomarkers. In addition, we describe potential biomarkers and genetic loci that have been discovered to be associated with psychiatric diseases and relevant treatments. Moreover, we outline the limitations in regard to the previous precision psychiatry and pharmacogenomics studies. Finally, we present a discussion of directions and challenges for future research.

## 1. Introduction

Precision psychiatry, which is an emerging interdisciplinary field of psychiatry, precision medicine, and pharmacogenomics, is developing into essential practices in medicine with a promise of the individualization of clinical care for patients with psychiatric disorders. It should be pointed out that pharmacogenomics is sometimes used interchangeably with precision medicine. In other words, pharmacogenomics is one of the research fields to further advance precision psychiatry, where pharmacogenomics is defined as the study of how genes and their functions can influence a person’s response to medications. In general, precision psychiatry means that medical decisions, treatments, and practices are adapted to specific patients with psychiatric disorders [1]. More specifically, the whole population of patients with psychiatric disorders are separated into various groups by specific biomarkers, where multi-omics and/or neuroimaging datasets are available to represent these specific biomarkers within the context of precision psychiatry. Therefore, medications can be tailored individually to each respective patient by using pertinent or proportionate genetic biomarkers and/or imaging attributes [1]. To date, there are progressively growing genetic biomarkers and imaging attributes that may contribute to the prognosis and treatment response for patients with psychiatric disorders [2]. For instance, it has long been recognized that genetic biomarkers such as gene expression profiles and single nucleotide polymorphisms (SNPs) can be utilized to evaluate adverse drug reactions and clinical treatment response for antidepressants in patients with major depressive disorder (MDD) [3,4].

In the field of precision psychiatry, researchers integrate multiple data types such as multi-omics and neuroimaging data with state-of-the-art artificial intelligence and machine learning algorithms, which can accordingly learn to identify complex patterns with respect to observational datasets [5,6,7]. Namely, multi-omics and neuroimaging data are employed to serve as biomarkers (or predictive factors) to fulfill the concept of precision psychiatry by using artificial intelligence and machine learning algorithms. In order to address the demanding challenges we face today in the field of precision psychiatry, there is an enormous need for developing software tools in artificial intelligence and machine learning frameworks that can predict specific quantitative and/or categorical phenotypes in clinical settings by utilizing next-generation multi-omics and neuroimaging datasets [5,6,7].

In recent times, scientists have been making significant progress in the multidisciplinary fields of artificial intelligence, machine learning, precision psychiatry, pharmacogenomics, multi-omics, and neuroimaging [5,6,7]. The goal of an artificial intelligence and machine learning approach is to provide a data-driven algorithm that can in general learn from the data in the past and/or in the present by leveraging the learned insight into estimating predictive outcomes for any unknown data and/or for any unknown event in the future [8,9,10]. In the general terms, the guideline of an artificial intelligence and machine learning approach is comprised of the following three steps: we firstly build the predictive model from the initial input data in the beginning step, then secondly fine-tune and gauge the predictive model in the intermediate step, and thirdly utilize the predictive model for presenting an estimated outcome in the final step [8,9,10].

Latest advancements in artificial intelligence and machine learning technologies, especially deep learning algorithms, have revealed their promising capacities to recognize and learn complex and nonlinear hierarchical patterns with respect to mammoth large-scale experimental data [11,12,13,14,15]. Furthermore, deep learning algorithms have accomplished state-of-the-art performances on a wide range of medical applications such as precision psychiatry based on recent new technologies such as the invention of general-purpose computing on graphics processing units [6,12,13,14,15]. Primarily, the objective of deep learning algorithms is to build artificial intelligence and machine learning algorithms which employ multiple layers of abstraction such as artificial neural networks to construct a hierarchical representation for the data [12,13,14,15,16]. That is, deep learning algorithms for classification applications, such as diagnosis prediction in precision psychiatry, are procedures for determining the best hypothesis by utilizing artificial neural networks with multiple layers, instead of utilizing artificial neural networks with only one single layer [12,13,14,15,16].

With the recent advance in multi-omics and neuroimaging technologies, precision psychiatry shows high growth potential to respond to the needs of new diagnostic tools as well as novel drugs for treatment and therapeutic interventions [17]. Furthermore, the usage of biomarkers has played a key role in precision psychiatry based on artificial intelligence and machine learning approaches [17]. In the recent past, there were a wide variety of emerging vital research studies for numerous diseases and treatments of significance for precision psychiatry with consideration of artificial intelligence and machine learning methods [6]. Accordingly, it could be remarkably intriguing to design artificial intelligence and machine learning algorithms that can predict the potential outcomes of drug treatments and disease status for patients with psychiatric disorders [6,17]. To address this challenge, artificial intelligence and machine learning approaches may yield helpful software tools to achieve the promise of precision psychiatry by concerning specific biomarkers for drug treatments and disease status [6,17]. In this review, we show recent research studies in precision psychiatry and pharmacogenomics, which assessed disease status and drug treatments using artificial intelligence and machine learning approaches, such as deep learning and artificial neural network algorithms. We mainly focus on multi-omics and neuroimaging data. Additionally, we present the limitations in these research studies and summarize a discussion of future challenges as well as directions.

Here, in the context of artificial intelligence and machine learning methods, we provide various research studies with focus on four major categories including treatment prediction, prognosis prediction, diagnosis prediction, and the detection of potential biomarkers in terms of psychiatric disorders, precision psychiatry, and pharmacogenomics. Biological and/or clinical implications from these four major arenas can serve as decision support aides for treatment prediction, prognosis prediction, and diagnosis prediction in translational and precision psychiatry [18]. Furthermore, we particularly focus on psychiatric disorders such as MDD, bipolar disorder, attention deficit hyperactivity disorder, Alzheimer’s disease, autism spectrum disorder, and schizophrenia. While this review does not support the full set of related research studies reported in the literature, it nonetheless describes a synthesis of those that can markedly influence public and population health-oriented applications in psychiatric disorders, precision psychiatry, and pharmacogenomics in the near to mid-term future.

## 2. Applications in Treatment Prediction

The usage of artificial intelligence and machine learning methods is still in its infancy in terms of forecasting drug treatments in psychiatric drugs due to the fact that scant human studies have explored the predictive models of evaluating drug treatment response. Here, we focus on antidepressant and lithium treatment outcomes using deep learning strategies as well as conventional artificial intelligence and machine learning methods in this section (Table 1). In this review, we first conducted a comprehensive search of the electronic PubMed database (2015-present) using key words such as “machine learning,” “deep learning,” “antidepressant,” “lithium,” “major depressive disorder,” “bipolar disorder,” and “pharmacogenomics”. Then, we manually screened the obtained articles with a particular focus on MDD. The multi-omics data in these selected studies included SNPs datasets, DNA methylation datasets, gene expression datasets, and phenotypic datasets (such as demographic and clinical datasets). In addition, the reader can refer to a recent review by Pisanu and Squassina [19] for treatment-resistant schizophrenia, where patients with treatment-resistant schizophrenia are defined as those revealing little or no response to at least two non-clozapine antipsychotic medications. The reader can also refer to a recent review by Perlman et al. [20] for predictors of antidepressant treatment response in MDD, where predictor categories include demographic factors, symptom profiles (such as age of onset), peripheral markers (accessible through urine, blood, or saliva), genetic biomarkers, and neuroimaging data.

To estimate potential individual-specific antidepressant treatment response in MDD, Lin et al. [21] investigated a deep learning architecture by leveraging an integrated data from various data types (encompassing genetic datasets such as SNPs, demographic datasets such as marital status, age, and sex, as well as clinical datasets such as suicide attempt status, baseline Hamilton Rating Scale for Depression score, and depressive episodes for MDD patients). The multi-omics data in their study included the SNPs, demographic, and clinical datasets). Firstly, Lin et al. [21] carried out a genome-wide association study (GWAS) to identify potentially significant associations of SNPs with antidepressant treatment response and remission in a hypothesis-free manner. Furthermore, the deep learning structure is comprised of multi-layer feedforward neural networks (MFNNs), which adapt the back-propagation algorithm. MFNNs were utilized to evaluate the probable complicated interplay between biomarkers and antidepressant treatment response [21]. Moreover, Lin et al. [21] revealed an MFNN predictive model with two hidden layers (area under the receiver operating characteristic curve (AUC) = 0.82; sensitivity = 0.75; specificity = 0.69) for antidepressant treatment response and another MFNN predictive model with three hidden layers (AUC = 0.81; sensitivity = 0.77; specificity = 0.66) for remission [21]. The benefit of this deep learning structure is that the MFNN predictive models have the advantages of integrated architectures, nonlinear representations, fault tolerance, and real-time processing [21].

To predict antidepressant treatment response, various research studies [22,23,24,25,26,27,28,29,30] used conventional artificial intelligence and machine learning methods. For example, Kautzky et al. [22] suggested that the random forest structure, a conventional artificial intelligence and machine learning method, correctly predicted 25% of responders for antidepressant treatment outcome based on genetic and clinical data. The random forest structure is an ensemble learning method, which is comprised of a multitude of decision trees for performing classification tasks [22]. The multi-omics data in their study included the SNPs and clinical datasets. In particular, Kautzky et al. [22] pinpointed some probable biomarkers such as a clinical variable known as melancholia as well as three SNPs such as the rs6265 SNP in the brain derived neurotrophic factor (*BDNF*) gene, the rs6313 SNP in the 5-hydroxytryptamine receptor 2A (*HTR2A*) gene, and the rs7430 SNP in the protein phosphatase 3 catalytic subunit gamma (*PPP3CC*) gene.

In addition, the subsequent study by Patel et al. [23] reported that an alternating decision tree structure, a conventional artificial intelligence and machine learning method, predicted antidepressant treatment response with an accuracy of 89% by using multiple datasets such as mini-mental status examination scores, age, and structural imaging. The multi-omics data in their study included the clinical and demographical datasets. The alternating decision tree structure, which generalizes decision trees, is relevant to boosting for scaling down variance and bias primarily [23].

On another note, Chekroud et al. [24] showed that a tree-based ensemble structure, a conventional artificial intelligence and machine learning method, predicted clinical antidepressant remission with an accuracy of 59% based on 25 variables [24]. The multi-omics data in their study included the clinical and demographical datasets. Firstly, the top 25 predictors was determined by the elastic net. Then, a tree-based ensemble method, also known as a gradient boosting machine algorithm, was employed to integrate various weak predictive decision trees to form a final ensemble structure [22]. Particularly, Chekroud et al. [24] found the top three probable biomarkers for antidepressant non-remission including reduced energy level during the past seven days, baseline depression severity, and feeling restless during the past seven days [24]. Moreover, the top three probable biomarkers for antidepressant remission include loss of insight into one’s depressive condition, total years of education, and currently being employed [24]. One strength of the tree-based ensemble structure is that predictors in the predictive model were externally validated by other independent cohorts [24].

Iniesta et al. [25] also demonstrated that an elastic net structure, a conventional artificial intelligence and machine learning method, can forecast antidepressant response with clinically meaningful accuracy (AUC = 0.72) based on clinical and demographical datasets. The multi-omics data in their study included the clinical and demographical datasets. The elastic net structure, also known as an application of regularized regression models, is a general linear model with penalties to implement the variable selection framework based on a large number of variables while avoiding overfitting [26].

Furthermore, a recent study by Maciukiewicz et al. [27] implicated that a support vector machine (SVM)-based and decision trees-based structure, a conventional artificial intelligence and machine learning method, can estimate antidepressant treatment response with an accuracy of 52% based on SNPs. The multi-omics data in their study included the SNPs datasets. Firstly, Maciukiewicz et al. [27] carried out a GWAS study to search for genetic susceptibility loci of antidepressant treatment response in a hypothesis-free manner. Then, least absolute shrinkage and selection operator (LASSO) regression was performed to find potentially significant predictors including the rs2036270 SNP in the retinoic acid receptor beta (*RARB*) gene and the rs7037011 SNP near the *LOC105375971* gene [27]. To strengthen the accuracy for treatment prediction, LASSO implements both regularization and variable selection [28].

In order to pinpoint the most competent antidepressant for a specific patient, Chang et al. [29] carried out an Antidepressant Response Prediction Network (ARPNet) model which employs useful features as predictors such as neuroimaging biomarkers, genetic variants, DNA methylation, and demographic information from the patient data. The multi-omics data in their study included the SNPs, DNA methylation, and demographical datasets. Moreover, the ARPNet model utilizes a linear regression method, a conventional artificial intelligence and machine learning approach, based on the extracted features from the patient records with an accuracy of 84% [29]. Their work suggested that the ARPNet model can help doctors prescribe the most effective antidepressant for their patients by computing the similarities between patients and by referring to the similar antidepressant response records of other patients [29].

Athreya et al. [30] employed a random forest method, a conventional artificial intelligence and machine learning approach, to predict antidepressant therapy response (with AUC > 0.7 and accuracy > 69%). The multi-omics data in their study included the SNPs datasets. Their approach was integrated with total baseline depression severity and six SNP biomarkers including the rs5743467, rs2741130, and rs2702877 SNPs in the defensin beta 1 (*DEFB1*) gene, the rs696692 SNP in the glutamate rich 3 (*ERICH3*) gene, the rs17137566 SNP in the aryl hydrocarbon receptor (*AHR*) gene, and the rs10516436 SNP in the tetraspanin 5 (*TSPAN5*) gene [30]. These six SNPs were identified as the top SNPs in the relevant GWAS findings.

Finally, various research studies [31,32] employed conventional artificial intelligence and machine learning methods to predict lithium treatment response in patients with bipolar disorder. For instance, Nunes et al. [31] reported that the random forest model, a conventional artificial intelligence and machine learning method, predicted responders for lithium treatment outcome in bipolar patients by using clinical data. The multi-omics data in their study included the clinical datasets. Their model implicated a clinically meaningful accuracy (AUC = 0.8; sensitivity = 0.53; specificity = 0.9) [31].

A recent study by Eugene et al. [32] also showed that the decision tree and random forest algorithms, conventional artificial intelligence and machine learning methods, predicted lithium responders in bipolar patients based on gene expression data (AUC = 0.92). The multi-omics data in their study included the gene expression datasets. In their work, the RNA binding protein with multiple splicing 2 (*RBPMS2*) and leukocyte immunoglobulin-like receptor subfamily A member 5 (*LILRA5*) genes were used for classifying male lithium responders [32]. On the other hand, ABRA C-terminal like (*ABRACL*), four and a half LIM domains protein 3 (*FHL3*), and NBPF member 14 (*NBPF14*) genes were employed to classify female lithium responders [32].

## 3. Applications in Prognosis Prediction

Several works have applied artificial intelligence and machine learning methods to predict future medical outcomes based on the current patient clinical and illness status. In this review, we first conducted a comprehensive search of the electronic PubMed database (2015–present) using key words such as “machine learning,” “deep learning,” “electronic health records,” “major depressive disorder,” “psychiatric disorder,” and “prognosis”. Then, we manually screened the obtained articles with a particular focus on psychiatric disorders such as MDD, attention deficit hyperactivity disorder, and schizophrenia. The multi-omics data in these selected studies included phenotypic datasets (such as demographic and clinical datasets).

For example, in order to identify three course trajectories of MDD (including chronic, gradual improving, and fast remission patients), Schmaal et al. [33] investigated a machine learning approach that employs the Gaussian process algorithm as a classifier to integrate functional and structural magnetic resonance imaging (MRI) data. Gaussian process classifiers, which are similar to SVMs, are a form of multivariate pattern recognition methods providing the support of predictive probabilities for class membership [33]. Their study used Gaussian process classifiers to assess three course trajectories of MDD by utilizing prognostic values of clinical datasets (including duration, comorbidity, and baseline severity) as well as MRI datasets [33]. Schmaal et al. [33] demonstrated that Gaussian process classifiers can distinguish chronic MDD patients from remitted MDD patients with an accuracy of up to 73%.

In order to facilitate clinical predictive modeling, Miotto et al. [34] proposed Deep Patient, which is a deep learning architecture constructing a general-purpose patient representation from electronic health records (EHRs), to predict health status and better inform clinical decision making. More precisely, Deep Patient employs multi-layer artificial neural networks, which represent a three-layer stack of denoising autoencoders. In order to derive dependencies and hierarchical regularities in the sample of more than 700,000 patients, the denoising autoencoders are equipped with sigmoid activation functions [34]. Deep Patient uses the random forest algorithm as classifiers to assess the probability that patients may develop a certain disease given their current clinical status [34]. To avoid overfitting, Deep Patient trains the denoising autoencoders to reproduce the input dataset from a noisy version of the original dataset [34]. It was indicated that Deep Patient can forecast psychiatric disorders such as attention deficit hyperactivity disorder or schizophrenia with high accuracy (AUC = 0.85) [34]. Furthermore, their study compared Deep Patient with raw EHRs or well-known conventional representation learning algorithms and found that the proposed deep learning architecture leads to significantly better performance metrics than those obtained by using only raw EHRs or well-known conventional representation learning algorithms such as Gaussian mixture model, principal component analysis, independent component analysis, and k-means clustering [34]. The major strength of Deep Patient is that the deep learning architecture can help pre-process the EHR data to yield more adequate predictions due to the effects of non-linear transformations [34].

Other similar software tools, such as DeepCare [35] and Doctor AI [36], also utilize EHRs and deep learning architectures to estimate future medical outcomes. Although these deep learning tools may not explicitly been applied to specific psychiatric disorders, it is still feasible to facilitate applications in precision psychiatry and precision medicine by using these software tools. DeepCare utilizes recurrent neural networks, which are equipped with long short-term memory hidden units [35]. Furthermore, DeepCare can handle irregular timed events in longitudinal EHRs [35]. Likewise, Doctor AI also employs recurrent neural networks, which are equipped with gated recurrent unit [36].

## 4. Applications in Diagnosis Prediction

Several works have applied artificial intelligence and machine learning methods to predict diagnosis of certain psychiatric disorders such as Alzheimer’s disease, autism spectrum disorder, and schizophrenia [37]. Nowadays, there has been a growing trend in combining artificial intelligence and machine learning techniques with neuroimaging (such as structural and functional neuroimaging) to provide new insights into psychiatric disorders such as schizophrenia, Alzheimer’s disease, and autism spectrum disorder [37]. In this review, we first conducted a comprehensive search of the electronic PubMed database (2015-present) using key words such as “machine learning,” “deep learning,” “neuroimaging,” “psychiatric disorder,” “Alzheimer’s disease,” “autism spectrum disorder,” “schizophrenia,” and “diagnosis”. Then, we manually screened the obtained articles with a particular focus on psychiatric disorders such as Alzheimer’s disease, autism spectrum disorder, and schizophrenia.

For instance, in order to discriminate normal aging from mild to severe sporadic Alzheimer’s disease, Kloppel et al. [38] pioneered a research project to use an artificial intelligence and machine learning method such as an SVM approach. Their study demonstrated that the predictive model achieved 89% accuracy based on structural MRI data [38]. Only MRI data and no multi-omics data were involved in their study. By using a two-step procedure, Kloppel et al. [38] used SVMs to learn the differences between healthy controls and patients with Alzheimer’s disease in the first process and then tested the predictive model with new brain scans in the second process.

Here, we particularly focus on emerging data science methodologies such as deep learning algorithms in the recent research studies. For example, in order to predict early diagnosis of Alzheimer’s disease, Ju et al. [39] reported a deep learning approach, which is comprised of a softmax regression layer and auto-encoders. Generally, an auto-encoder is an encoding structure, which is comprised of an artificial neural network with the input layer serving the MRI data, with multiple hidden layers serving nonlinear transformations from the previous layers, and with the output layer serving the reconstructed MRI samples. Only MRI data and no multi-omics data were involved in their study. Ju et al. [39] revealed that the proposed auto-encoder structure had a better accuracy (87.76%) when compared to widely-used single-kernel SVM (accuracy = 84.40%) and multi-kernel SVM (accuracy = 86.42%). Their work indicated that deep learning algorithms have an advantage over traditional artificial intelligence and machine learning methods for predicting and preventing Alzheimer’s disease at an early stage [39].

To identify Alzheimer’s disease, Ortiz et al. [40] also integrated MRI data with a deep learning structure, which is a belief network-based algorithm combining the automated anatomical labeling data with the gray matter images of brain regions. Only MRI data and no multi-omics data were involved in their study. In the field of artificial intelligence and machine learning, a deep belief network is a class of deep artificial neural networks comprising of multiple layers of latent variables and serving as a composition of auto-encoders [41]. Ortiz et al. [40] reported that the deep learning structure consists of an ensemble of two deep belief networks with four different voting schemes. Their study revealed that the proposed deep belief network method had good performances for distinguishing samples between Alzheimer’s disease and healthy controls (accuracy = 90%) as well as for distinguishing samples between Alzheimer’s disease and mild cognitive impairment (accuracy = 84%) [40].

Moreover, deep belief networks have been used to characterize differences between healthy controls and young children with autism spectrum disorders based on functional MRI data [41]. Additionally, Pinaya et al. [42] employed a deep belief network predictive model to differentiate healthy controls from patients with schizophrenia (accuracy = 73.6%) based on MRI data. Only MRI data and no multi-omics data were involved in their study. Interestingly, the performance for the deep belief network predictive model (accuracy = 73.6%) was better than the SVM predictive model (accuracy = 68.1%) [42].

In a recent study, Lin et al. [43] explored artificial intelligence and machine learning tools to distinguish schizophrenia patients from healthy controls by leveraging SNPs and protein levels in the D-amino acid oxidase activator (*DAOA*, also known as *G72*) gene. The multi-omics data in their study included the SNPs, protein, demographic, and clinical datasets). Their work utilized logistic regression, naive Bayes, and C4.5 decision tree to build predictive models [43]. Lin et al.’s results revealed that the naive Bayes model using the rs1421292 SNP and protein levels in the *G72* gene was the best predictive model for distinguishing schizophrenia patients from healthy controls (AUC = 0.9356; sensitivity = 0.7969; specificity = 0.9372) [43].

## 5. Detection of Potential Biomarkers

Several works have applied artificial intelligence and machine learning methods to predict potential biomarkers in certain psychiatric disorders, such as pattern similarity scores for Alzheimer’s disease. In this review, we first conducted a comprehensive search of the electronic PubMed database (2015-present) using key words such as “machine learning,” “deep learning,” “psychiatric disorder,” “Alzheimer’s disease,” and “detection of potential biomarkers”. Then, we manually screened the obtained articles with a particular focus on Alzheimer’s disease.

For example, in order to pinpoint a new risk biomarker called Alzheimer’s disease pattern similarity scores, Casanova et al. [44] carried out a high-dimensional artificial intelligence and machine learning architecture which contains a regularized logistic regression algorithm with an elastic net. This high-dimensional architecture can concurrently achieve prediction for Alzheimer’s disease as well as data integration [44]. The regularized logistic regression algorithm with the elastic net, also known as the adaptive elastic net, has been successfully applied to high-dimensional datasets [26,45]. In the predictive model, the adaptive elastic net employs elastic net estimates as the initial weight [26,45]. The neuroimaging data (such as MRI datasets) and multi-omics data (such as demographic and clinical datasets) were involved in their study, where the MRI datasets incorporate the Alzheimer’s Disease Neuroimaging Initiative (ADNI) database. The approach by Casanova et al. [44] was integrated with the neuroimaging data from the ADNI baseline structural MRI datasets and can distinguish patients with Alzheimer’s disease from healthy controls based on a coordinate-wise descent technique [46]. Casanova et al. [44] suggested that Alzheimer’s disease pattern similarity scores were strongly associated with cognitive status, age, and cognitive function and thereby can serve as a risk biomarker for Alzheimer’s disease.

To our knowledge, no other recent studies have investigated artificial intelligence and machine learning methods for the detection of potential biomarkers in Alzheimer’s disease.

## 6. Limitations

The discoveries as illustrated in the aforementioned sections should be explained by taking into account various disadvantages of these research studies in the interdisciplinary fields of precision psychiatry, pharmacogenomics, artificial intelligence, machine learning, multi-omics, and neuroimaging. One disadvantage of these previous studies is that the small-scale sample sizes do not allow for contributing to well-defined conclusions because of the risk of overfitting during the training step of artificial intelligence and machine learning algorithms [47]. Secondly, it is crucial to generalize to independent cohorts for these associations by employing worldwide populations [6]. Yet, most of these research studies did not use replication data since large-scale data might not be accessible for facilitating subsequent analysis. Therefore, these discoveries might not be generalizable. An open challenge is that in the most proper discoveries, we would generalize to independent cohorts with various underrepresented ethnic groups, different testing conditions, diverse recruitment sites, numerous real-life clinical settings, and broader worldwide populations [48]. Thus, future predictive models should be reliably reproducible to operate on new individual cases across diverse metrics, populations, geographic locations, clinical settings, comorbidity profiles, and data-acquisition methods [6].

While an aforementioned predictive model was built based on a particular antidepressant (for example, citalopram/escitalopram), this specific model was designed to predict the response of only one antidepressant and thereby may not be replicated for other antidepressants [30]. Thus, it is pivotal for future predictive models to possess the ability of generalizing and predicting a generic treatment response. Moreover, a common pitfall is that the aforementioned predictive models may not be transparent enough to interpret relationships between biomarkers (predictive factors) and their predictions [18]. Another limitation is between-site heterogeneity in the data due to data collection in several different recruitment sites [31]. In addition, a potential flaw of the analysis in some studies is that the inclusion of patients with comorbid general medical conditions might have influenced drug treatment outcome [30].

Yet another pitfall is that it is particularly challenging to handle longer-term disease trajectories for most of aforementioned predictive models because those predictive models were typically based on retrospective data instead of longitudinal data [6]. While retrospective datasets could be generally more applicable for cross-sectional analysis, prospective (or longitudinal) datasets could be more useful for future predictive models to deal with a time-varying nature of various psychiatric disorders/diseases [6]. It is also worth mentioning that proper statistical harmonization methods must be performed prior to utilizing future predictive models to handle large high-dimensional neuroimaging datasets because of data heterogeneity [7]. That is, before performing downstream analyses for neuroimaging data, various statistical harmonization techniques such as functional normalization and surrogate variable analysis can be employed to minimize or remove unwanted data variation induced by data collection from multiple sites [7].

Furthermore, it should be pointed out that variability in data quality such as missing data could make the pre-selection of driver biomarkers and/or predictive variables indispensable. For instance, prior-selecting SNPs from the GWAS datasets could be a good procedure [49]. Nonetheless, we hypothesize that the prior-selection may be supposed to influence the long-term performance and architecture in the eventual artificial intelligence and machine learning framework. In future studies, large prospective clinical trials are warranted such that we could pinpoint whether the related biomarkers are generalizable to be linked to drug treatments and/or disease status for research studies in precision psychiatry.

Although in the present review we showed some research reports to depict the related artificial intelligence and machine learning algorithms in the neurobiology of psychiatric disorders, it might be noted that precision psychiatry studies may be further improved by integrating the state-of-the-art artificial intelligence and machine learning frameworks with multi-omics and neuroimaging datasets [17]. Numerous current biobanks have been built to collect multi-omics data, such as the Taiwan Biobank [50,51,52,53,54,55,56,57], the COMBINE Biobank [58], LifeGene [59], and the Korea Healthy Twin Study [60].

Certainly, it should be mentioned that future research in precision psychiatry and pharmacogenomics for psychiatric disorders could employ Venn diagrams (also called set diagrams) to examine both the overlap and lack of overlap among biomarkers in different artificial intelligence and machine learning algorithms so that the stability of one single biomarker and/or even a group of multiple biomarkers could be shown [61]. That is, we could use Venn diagrams to show all possible logical relations among biomarkers in various artificial intelligence and machine learning algorithms. Ongoing evidence suggests that different artificial intelligence and machine learning algorithms might usually generate diverse biomarkers, even when the algorithms were provided with the equivalent form of drug treatments and/or diseases [62].

## 7. Other Relevant Studies in Psychiatric Disorders

In the previous sections, we mention a wide variety of research studies in precision psychiatry with focus on various psychiatric disorders including MDD, bipolar disorder, attention deficit hyperactivity disorder, Alzheimer’s disease, autism spectrum disorder, and schizophrenia. While this review does not intend to cover all psychiatric disorders/diseases that have been studied in an exhaustive manner, it nevertheless is representative of the general trend for current research in precision psychiatry using artificial intelligence and machine learning approaches. However, it is arguable that what psychiatric disorders/diseases could likely be considered as the focus of attention in the field of precision psychiatry nowadays. For other relevant research studies in precision psychiatry using artificial intelligence and machine learning approaches, the reader can refer to a recent review by Goldstein-Piekarski et al. [63] for sleep impairment in mood and anxiety disorders, a recent review by Mak et al. [64] for substance addiction (for example, smoking addiction and alcohol addiction) and non-substance addiction (for instance, gambling addiction and internet addiction), as well as a recent review by Ramos-Lima et al. [65] for trauma-related disorders such as acute stress disorder and post-traumatic stress disorder.

For example, Goldstein-Piekarski et al. [63] indicated that artificial intelligence and machine learning approaches could extract authentic sleep features related to sleep impairment in mood and anxiety disorders, where sleep disorders, mood disorders, and anxiety disorders often occur as comorbid illnesses. In addition, artificial intelligence and machine learning approaches could be applied to identify a meaningful combination of sensor data that are associated with sleep impairment in mood and anxiety disorders, where the sensor data (collected from sensing and wearable technologies) consist of accelerometry counts, skin temperature, phone calls, text messages, web usage, and email usage [63].

On another note, Mak et al. [64] suggested that artificial intelligence and machine learning approaches could help combine the assessment scale data with the medical testing data to predict substance and non-substance addiction, with the majority being in research studies of addictive behaviors in cigarette smoking, alcohol drinking, and cocaine use. While substance addiction covers nicotine, alcohol, cocaine, and marijuana addiction, non-substance addiction (also known as behavioral addiction) includes internet addiction, pathological gambling, mobile device addiction, and food addiction [64].

Furthermore, Ramos-Lima et al. [65] implicated that new discovery for trauma-related disorders such as acute stress disorder and post-traumatic stress disorder could be advanced through artificial intelligence and machine learning approaches in diverse populations (for instance, disaster survivors, military veterans, and refugees in war zones), where traumatic symptoms encompass persistent negative thoughts or feelings, avoidance, re-experience of the traumatic event, and trauma-related reactivity and arousal as listed in diagnostic criteria.

## 8. Conclusion and Perspectives

As indicated by the aforementioned findings, precision psychiatry affirms to provide novel diagnostic and therapeutic approaches for treatment prediction, prognosis prediction, diagnosis prediction, and the detection of potential biomarkers in an individual-specific and treatment-specific manner in psychiatric disorders [1,2,66,67]. In terms of neuroimaging-driven and multi-omics-driven techniques, it is of great interest that future prospective clinical trials concerning artificial intelligence and machine learning approaches to forecast medical outcomes and/or drug treatments may contribute to feasible explanations in public health as well as global health. Thus, governments and the general public should consider these issues and challenges with a high priority. Thereby, we expect that therapies for psychiatric disorders over the next few years must take into consideration of the interactions between multi-omics and neuroimaging datasets as well as gene–environment interactions and epigenetics [68,69,70]. The recent advancements in data-intensive health sciences and single cell sequencing technologies could assuredly trigger new artificial intelligence and machine learning software frameworks, such as deep learning algorithms [71], for population health, public health, and global health in the up-coming decade [72,73]. Furthermore, individual-oriented results will be progressively generated towards the fields of population health, public health, and global health in light of the pressing needs of innovative diagnostics in precision psychiatry and pharmacogenomics for psychiatric disorders [74,75]. In the next generation, the pre-treatment prediction tests involving artificial intelligence and machine learning-based precision psychiatry and pharmacogenomics would become a reality in individual-specific clinical care when prospective large-scale studies are able to assess thoroughly the related biomarkers as well as clinical factors [76,77].

## Figures and Tables

**Table 1:** Relevant studies on the predictive models of evaluating drug treatment response.

Study	Model	Results
Lin et al. [21]	Deep learning architecture	AUC = 0.82, sensitivity = 0.75, specificity = 0.69 for antidepressant treatment response; AUC = 0.81, sensitivity = 0.77, specificity = 0.66 for remission
Kautzky et al. [22]	Random forest	An accuracy of 25% for antidepressant treatment outcome
Patel et al. [23]	Decision tree	An accuracy of 89% based on mini-mental status examination scores, age, and structural imaging
Chekroud et al. [24]	Tree-based ensemble	An accuracy of 59% based on 25 variables for clinical antidepressant remission
Iniesta et al. also [25]	Elastic net	AUC = 0.72 based on clinical and demographical datasets
Maciukiewicz et al. [27]	SVM and decision trees	An accuracy of 52% based on SNPs
Chang et al. [29]	Linear regression	An accuracy of 84% based on neuroimaging biomarkers, genetic variants, DNA methylation, and demographic information
Athreya et al. [30]	Random forest	AUC > 0.7 and accuracy > 69% for antidepressant therapy response
Nunes et al. [31]	Random forest	AUC = 0.8; sensitivity = 0.53; specificity = 0.9 for lithium therapy response
Eugene et al. [32]	Decision tree and random forest	AUC = 0.92 for lithium therapy response

AUC = area under the receiver operating characteristic curve; SNPs = single nucleotide polymorphisms; SVM = support vector machine.
